# Micro-Photographs in Histology—Normal and Pathological

**Published:** 1876-10

**Authors:** 


*Micro-Photographs in Histology, Normal and Pathological.*—By Carl Seiler, M. D., in conjunction with J. Gibbons Hunt, M. D., and Jos. G. Richardson, M. D.

This publication, of which we have received the first five numbers, is a beautiful specimen of art, intended to replace the microscope as far as possible for those who have not opportunity or time to use this instrument. It furnishes correct representations of typical specimens of normal and pathological histology for comparison and for use in the lecture room, as illustrations.

The pictures are necessarily faithful copies and exact impressions of the specimens, as they are obtained directly from micro scropic objects by means of photography, and printed from the negative by a reliable mechanical process. The process employed in preparing these pictures insures sharpness and distinctness in the greatest degree, and each plate is accompanied by several pages of text fully describing it, and showing the connection and analogy of the different specimens, being strictly descriptive and explanatory, and having no reference to unacknowledged theories. Every monthly issue will contain at least one pathological and three normal specimens to illustrate the differences between health and diseased structures, thus offering clear insight into the nature of the pathological formations and structures as revealed by the microscope. This admirable and useful work is issued in monthly numbers, twelve to form a volume, at 60 cents each or $6.00 per annum, and is the only publication of this kind. Publishers, J. H. Coates & Co., 822 Chestnut Street, Philadelphia.

---

